# Supplementary material for: One dimensional approximations of neuronal dynamics reveal computational strategy
Source: PLoS Comput Biol. 2023 Jan 6;19(1):e1010784. doi: 10.1371/journal.pcbi.1010784 (PMC9821456; doi:10.1371/journal.pcbi.1010784)
Supplement: S1 Text — Contains details of all algorithms used in the LOOPER method, along with details of analysis used in the Main Text. Also includes Supplementary Figures. (PDF) [file pcbi.1010784.s001.pdf]

Supplement

Here we provide a step by step mathematical description of LOOPER.

0.1 Step 1: Asymmetric diffusion mapping

In everything that follows, we assume that the data are in state space. In this section we describe how diffusion maps are used to linearize the dynamics. Diffusion mapping is a nonlinear dimensionality reduction technique. Like other nonlinear dimensionality reduction techniques, diffusion maps utilize the fact that nonlinear manifolds are locally Euclidean. In diffusion maps, distances between points in a local neighborhood are expressed as transition probabilities using a diffusion kernel where the probability of a point, $\mathbf{x}_1$, transitioning to another point, $\mathbf{x}_2$ in one time step is:

$$P(\mathbf{x}_1, \mathbf{x}_2) = exp\left(-\frac{D(\mathbf{x}_1, \mathbf{x}_2)^2}{2\sigma^2}\right), \tag{A}$$

where D is a distance measure and σ is a normalization term that sets the size of the local neighborhood.

$$P_{local}(\mathbf{x}_i, \mathbf{x}_j) = \begin{cases} P(\mathbf{x}_i, \mathbf{x}_j) & \text{if } \mathbf{x}_j \in nbh(\mathbf{x}_i) \\ 0 & \text{elsewhere} \end{cases} \tag{B}$$

where $nbh(\mathbf{x}_i)$ is the local neighborhood of $\mathbf{x}_i$ and is defined as all points, $\mathbf{x}$ such that $D(\mathbf{x}_i, \mathbf{x}) < 2\sigma$.

The pairwise local transition probabilities between all n points are assembled into a $n \times n$ global transition probability matrix by applying P_{local} to each of the n experimental observations and normalizing such that the matrix is right stochastic. This matrix models diffusion with a local neighborhood after one time step. Exponentiation of this transition probability matrix yields diffusion over longer time periods. In this way the global nonlinear structure of the dynamics can be stitched together from local neighborhoods.

Before Eq. SB can be applied to the data to assemble the transition probability matrix, distance measure D and an appropriate scaling for the neighborhood σ have to be defined. The methodology used to accomplish these goals is described below.

0.2 Distance

We are specifically interested in modeling dynamical systems. In order to account for the temporal sequencing of the data, we take both the location of the data and its empirically observed time derivative (velocity) into account when computing distances between points:

$$D_{\text{combine}} = 1 - (1 - P_{D_{\dot{x}}}) \cdot (1 - P_{D_{\mathbf{x}}}). \tag{C}$$

where $P_{D_{\dot{x}}}$ is the normalized difference in velocity, and $P_{D_{\mathbf{x}}}$ is the normalized difference in position. Differences in position $D_{\mathbf{x}}$ and velocity $D_{\dot{\mathbf{x}}}$ are normalized as follows:

$$P_D = D(\mathbf{x}_1, \mathbf{x}_2) / \max_{\mathbf{x}} D(\mathbf{x}_1, \mathbf{x}), \tag{D}$$

where D is one of the two distance measures as above, and the denominator is the maximum value the distance takes over all observed points. For the difference in position we use standard Euclidean distance scaled by the approximation of the local noise (see section **Estimation of Local Noise**). For difference in velocity (also scaled by the approximation of the local noise) we use the cosine distance:

$$D_{\dot{\mathbf{x}}}(\mathbf{x}_i, \mathbf{x}_j) = 1 - \frac{\dot{\mathbf{x}}_i \cdot \dot{\mathbf{x}}_j}{|\dot{\mathbf{x}}_i|\,|\dot{\mathbf{x}}_j|}, \tag{E}$$

where $\cdot$ is the inner product and $|\cdot|$ is the magnitude of the vector. The velocity is the distance between $\dot{\mathbf{x}}_i$ and the next observed time point. Note that $D_{combine}$ is small only when two points $\mathbf{x}_i$ and $\mathbf{x}_j$ are near each other and move in the same direction. Thus, $D_{combine}$ is a natural Newtonian way of defining distances between experimental observations that arise in dynamical systems.

0.3 Partitioning the data into local neighborhoods

One of the greatest challenges for constructing a diffusion map is selecting the correct size for the local neighborhood in Eq. SB. In this section we describe how this is accomplished in LOOPER. Once the local neighborhood is defined, it has to be rescaled in order to take into account anisotropic diffusion. This rescaling will be addressed in section titled **Estimation of Local Noise**.

One strategy for defining a local neighborhood is to use k-nearest neighbors. An alternative approach sets the size of the point cloud and assigns all the points inside this cloud to a local neighborhood. Choosing appropriate k, or the appropriate neighborhood size, however, is not trivial. This choice is made harder still because there is

no guarantee that the optimal k, or neighborhood size is the same everywhere. One approach is to choose a different optimal k for each neighborhood. This approach can work but quickly becomes computationally prohibitive. Thus, here we utilized a more computationally efficient strategy that first uses k-nearest neighbor approach to estimate local noise and then uses this estimate of noise to define the size of the local neighborhood.

First, we define a single k for all neighborhoods. This is a free parameter of the method. The primary purpose of choosing k is to select a subset of data which will be used to compute the local noise σ_{local}.

We start with computing the Euclidean distances from the point of interest $\mathbf{x}_t$ to all other data points. The naive approach would be to simply find k-nearest points. However, if the experimental observations are sampled finely enough in time, points in the neighborhood of $\mathbf{x}_t$ will most likely be observed around time t. We are interested in recurrent trajectories – dynamics repeatedly observed during execution of the task. To find nearest trajectories, rather than just nearest points, we utilize an approach similar to constructing return maps [1] – a dynamical systems methodology which identifies states to which the dynamical system returns multiple times. Thus, we define a minimum return time, τ (a parameter in the method). τ should be sufficiently large to assure that the system first leaves the neighborhood of $\mathbf{x}_t$ prior to returning to it. The first nearest neighbor is the closest point that is separated in time from the point of interest by at least τ. The second nearest neighbor is the next closest point that is separated in time from both the point of interest and the first nearest neighbor by at least τ. This iterative process is repeated until k nearest neighbors have been selected. The result of this approach is a collection of points that together form a local neighborhood to which the system returns during repeated execution of behaviors. We define this set of points as $\mathcal{N}_{original}(\mathbf{x}_t)$. This subset of points will be now used to estimate local noise σ_{local} around each experimental observation as described in the following section.

0.4 Estimation of Local Noise

In general the stability of the dynamics do not need to be isotropic. This implies that the local noise may also differ along different dimensions. To account for this anisotropy, the distances between $\mathbf{x}_t$ and all other points have to be rescaled. We construct an agglomerate neighborhood by combining $\mathcal{N}_{original}(\mathbf{x}_t)$ and all points that are one time step removed (either preceding or following) each point within $\mathcal{N}_{original}(\mathbf{x}_t)$. The result is a $3k$-sized point cloud. We then estimate the variance of this cloud along each dimension, σ_{local}. Note that we choose to ignore covariance as the number of observations for each local neighborhood can be small relative to the number of dimensions of the data. For N-dimensional data, σ_{local} is an N-dimensional vector $\left\langle \sigma_{local}^{(1)}, \sigma_{local}^{(2)} \ldots \sigma_{local}^{(N)} \right\rangle$.

This vector is used to rescale distances between the point of interest $\mathbf{x}_t$ and all other points as follows:

$$D_{\mathbf{x}}(\mathbf{x}_t, \mathbf{x}) = \sqrt{\sum_{i=1}^{N} \left(\frac{x_t^{(i)} - x^{(i)}}{\sigma_{local}^{(i)}} \right)^2}, \quad \text{(F)}$$

where $x^{(i)}$ is the i-th dimension of the N-dimensional data and $\sigma_{local}^{(i)}$ is the i-th dimension of the local noise. This equation corresponds to expressing Euclidean distances between the point of interest $\mathbf{x}_t$ and all other points in terms local z-scores. This normalization approximately spherizes local neighborhoods and corrects for anisotropy in the local stability. Because σ_{local} is computed locally for each data point, this normalization allows for changing stability in the trajectory as it traverses state space. We apply conceptually the same normalization to the cosine distance of velocities given in Eq. SE. The two distance measures used in Eq SE and Eqs SF have now been defined and can be used to compute $D_{combine}$ (Eq. SC).

The final step in constructing the local neighborhood is to choose the normalization term, σ, for Eq. SA which ultimately limits the size of the diffusion kernel. To accomplish this we apply the same k-nearest neighbor approach as in section **Partitioning the data into local neighborhoods** to construct $\mathcal{N}_{scaled}(\mathbf{x}_t)$, using the distance measure $D_{combine}$. We then choose σ to be equal to the largest distance between $\mathbf{x}_t$ and elements of $\mathcal{N}_{scaled}(\mathbf{x}_t)$. We now have assembled all the elements necessary to compute the diffusion map (Eq. SB). This map can be used to simulate diffusion after one time step. In the next section, we describe how global structure of the dynamics can be reconstructed from local transition probabilities.

0.5 Reconstruction of Global Distances

In order to increase the robustness of the local neighborhood we symmetrize the diffusion probabilities as follows:

$$P_{\text{final}}(\mathbf{x}_1, \mathbf{x}_2) = \min\left[P_{\text{local}}(\mathbf{x}_1, \mathbf{x}_2), P_{\text{local}}(\mathbf{x}_2, \mathbf{x}_1)\right] = P_{\text{final}}(\mathbf{x}_2, \mathbf{x}_1). \quad \text{(G)}$$

This allows the global information encoded in pairwise similarities to error check cases in which the local neighborhood estimate of a single point is poorly conditioned.

The diffusion map, L, can now be built by taking P_{final} for each pair of points in the observed dataset:

$$L_{ij} = P_{\text{final}}(\mathbf{x}_i, \mathbf{x}_j). \quad \text{(H)}$$

L only considers local transition probabilities after one time step. In order to explore the global structure of the dynamics, we must allow the system to evolve in time and diffuse beyond the local neighborhoods. This is

accomplished by first converting L to a Markov matrix:

$$L_{ij}^{\text{markov}} = \frac{L_{ij}}{\sum_j L_{ij}}. \tag{I}$$

We can then simulate the time evolution of the system by exponentiating this matrix. The goal of the exponentiation is to repopulate the elements beyond local neighborhoods. Thus, we exponentiate L^{markov} until the number of non-zero elements in a row of L^{markov} is greater than 95% (the "repopulation density" parameter of the method). Thus, through exponentiation, local neighborhoods are stitched together and global structure of the data is now encoded in:

$$L^{global} = (L^{markov})^T, \tag{J}$$

where T is the number of exponentiations required to repopulate the matrix.

We now normalize L^{global} to obtain a discrete approximation of the Fokker-Planck operator. The Fokker-Planck operator describes the time evolution of a distribution of points driven by a stochastic dynamical system. First, we approximate the steady state distribution, π, of the system using standard spectral analysis of L^{global}. Next, we normalize each element of L^{global} by the steady state distribution,

$$L_{ij}^{FP} = \frac{L_{ij}^{global}}{\sqrt{\pi_i \pi_j}}. \tag{K}$$

The resulting matrix is then converted to a Markov matrix, M, as before. M is the discrete Fokker-Planck operator of the system (Fig 1C) [2] which quantifies diffusion after T time steps.

Unfortunately, standard diffusion maps are not well suited for the extraction of coherent trajectories because they are symmetric by construction. Thus, M will always have the detailed balance condition, and describe purely diffusive processes. We are ultimately interested in finding coherent trajectories that may have non-zero flux. The only flux that satisfies the stationarity assumption is divergence free and cyclic [3]. Such fluxes correspond to rotations in state space. These rotations occur when the transition probability matrix has complex eigenvalues. Conceptually, this suggests a modification of the standard diffusion kernel by centering the kernel not on a point, $\mathbf{x}_t$, but on its next observed point, $\mathbf{x}_{t+1}$,

$$P(\mathbf{x}_t, \mathbf{x}) = exp\left(-\frac{D(\mathbf{x}_{t+1}, \mathbf{x})}{2\sigma^2}\right). \tag{L}$$

To achieve this, we remove the first column and last row of M. This is akin to shifting the transition probabilities to the upper one-off diagonal, and has the effect of making the most likely transition from the

current time point, $\mathbf{x}_t$, always be the next observed time point, $\mathbf{x}_{t+1}$. The resulting asymmetric matrix is a discrete approximation of a stochastic dynamical system that contains both diffusion and flux. By reducing this matrix we will ultimately be able to approximate the dynamics of the system. The next step in the LOOPER algorithm involves simplifying such matrices.

0.6 Step 2: Reducing the matrix

We begin by renormalizing the diffusion map so that similar points are grouped together. Thus, the overall goal of the next step is to convert the $n \times n$ matrix M spanned by experimental observations to a smaller $C \times C$ matrix where each element corresponds to a cluster of similar points.

The goal of the matrix renormalization is to condense the asymmetric diffusion map into a minimal number of states without destroying the information contained in the original matrix. The optimal number of states can be found using the minimum description length method [4], which balances the number of parameters of the model and the information loss of the model:

$$\mathrm{MDL} = I_{\mathrm{loss}} + \frac{k}{2} \log \frac{n}{2\pi}, \tag{M}$$

where I_{loss} is how much worse the new model, $M_{reduced}$, performs compared to the original model M, k is the size of $M_{reduced}$ and n is the size of M. $M_{reduced}$ is defined as follows:

$$M_{reduced}(i, j) = \sum_{a \in c_i, b \in c_j} \frac{M(a, b)}{|c_i|}. \tag{N}$$

Where a, b are indices of data points in M, c_i is the i-th cluster of points in $M_{reduced}$, and $|\cdot|$ is the number of elements in the cluster. Thus, the reduced matrix has $k = C^2$ elements, where C is the number of clusters in $M_{reduced}$. Further, every datapoint indexed by a of M maps to a cluster c_a in $M_{reduced}$ by $a \mapsto c_a$. To determine how much information is lost by such clustering, we reconstruct the original matrix from its clustered version.

$$M_{reconstruct}(a, b) = M_{reduced}(c_a, c_b). \tag{O}$$

The information lost in this $M_{reconstruct}$ is used to optimize the number of clusters in $M_{reduced}$.

We wish to enforce low information loss over all observed states. For computational efficiency, we constrain the upper bound of information loss and consider the states with the worst information loss. Thus, we approximate the information loss due to clustering of data points as:

$$I_{\mathrm{loss}} \approx n \cdot \max_t Q_{\mathrm{KL}}(0.95), \tag{P}$$

where Q_{KL} is the 95% quantile of the following value over all observed starting states, i:

$$D_{\mathrm{KL}}\left(M(t|t_0=i)\|M_{reconstruct}(t|t_0=i)\right). \tag{Q}$$

Here D_{KL} is the Kullback-Leibler divergence (a measure of how much information about the distribution is lost by applying a given approximation), $M(t|t_0=i)$ is the time evolution of the distribution over all observed states when starting from state i, and $M_{reconstruct}(t|t_0=i)$ is the time evolution of the distribution over observed states of the reconstructed matrix when starting from state i. The time evolution of a Markov matrix starting from state i is given by:

$$P(t|t_0=i)=P^t\cdot e_i \tag{R}$$

Where e_i is a vector with a 1 for the i-th element and zeros everywhere else. Note that i refers to the observed state, and not the corresponding reduced cluster. We maximize I_{loss} over time in order to account for the possibility that the reduced approximation can have initially low information loss that grows over time. This can occur when two clusters that are close in state space, but are in two different stable trajectories, are merged. In order to keep the computation tractable, we test t from 1 to 5 (with the option to test more values of t as needed). We then pick the number of clusters such that MDL is minimized over a range of putative cluster counts.

To compute putative $M_{reduced}$, we merge clusters in a hierarchical fashion using a similarity measure between states within the original matrix M. This similarity is defined as follows:

$$S_{ij}=1-\mathrm{corr}(M_i,M_j), \tag{S}$$

where M_i is the i-th row of the original diffusion map M. Intuitively this means that if the distribution of states observed in the next time step starting from state i and j is similar, then states i and j are similar. Hierarchical clustering based on S was implemented using the MATLAB linkage command. The end result of this procedure is a new $C\times C$ transition probability matrix that instead of experimental observations contains cluster ids as its entries (Fig SI). This renormalization tremendously simplifies discovery of loops described in the next section.

0.7 Step 3: Loop Finding

The objective in this step is to further simplify the $C\times C$ matrix $M_{reduced}$ by describing the dynamics that arise in it as a collection of interconnecting loops. The dynamics given by $M_{reduced}$ is a sequence of cluster ids. A loop, therefore is a sequence of cluster ids that begins and ends in the same cluster. We can conceptualize such a loop as a word, W, where each letter corresponds to a cluster id. Therefore, a natural way to compare different W is

to use an edit distance. We use this general approach in LOOPER. We first identify W given by the $M_{reduced}$. We then apply an edit distance to combine such W into loop clusters C_W. The clustering procedure is then optimized. The specific procedure for accomplishing this clustering and optimization is outlined below.

0.8 Finding Loops

We start with $M_{reduced}$ – a $C \times C$ diffusion map where each entry corresponds to a cluster, c, formed by the experimental observations. Using this information, we construct a bandwidth matrix B where each entry is the number of times that a data point observed to be in c_i at time t is found in c_j at time $t+1$:

$$B_{i,j\neq i} = \left|\{\mathbf{x}_t \in c_i, \mathbf{x}_{t+1} \in c_j\}\right|, \tag{T}$$

where $|\cdot|$ is the number of elements in the set and $\mathbf{x}$ is an experimental observation.

We next find the shortest path starting and ending in c_i – the shortest loop – using a standard Dijskstra shortest path algorithm [5] applied to $1/B$, where the inverse is element-wise. Thus, we find C shortest loops W, one for each cluster c. Clearly, some of these loops will overlap. For example, if a sequence of clusters $\{c_1, \ldots, c_n\}$ is a loop, then any shift permutations of this sequence is also a loop. In order to quantify similarity between two loops, we therefore employ an edit distance. However, the standard application of an edit distance cannot take into account the fact that some clusters are more similar to each other than others. Thus, prior to the application of an edit distance, similarity between clusters needs to be defined. This is accomplished as follows:

$$S_{\text{cluster}}(i,j) = \frac{M_{\text{cluster}_i} \cdot M_{\text{cluster}_j}}{\left\|M_{\text{cluster}_i}\right\| \left\|M_{\text{cluster}_j}\right\|} \tag{U}$$

where $\|\cdot\|$ is the Euclidean norm, and M_{cluster_i} is ith row of the $C \times n$ matrix of mean transition probabilities computed across all points that belong to a given cluster c_i to all n experimental observations:

$$M_{\text{cluster}_i} = \frac{1}{|c_i|} \sum_{t \in \mathrm{c}_i} M_t, \tag{V}$$

where $|\cdot|$ is the number of elements, and M_t is the tth row of M. $S_{cluster}(i,j)$ is analogous to S (Eq. SS) in that it quantifies the similarity in transition probabilities of all states that make up cluster i and cluster j. Now, equipped with the cluster similarity measure, we can readily compare loops, W formed by sequences of clusters as follows:

$$S_{\text{loop}}(i,j) = \prod_{c_k \in W_i} \max_{c_l \in W_j} S_{\text{cluster}}(c_k, c_l) \tag{W}$$

Note that when the same cluster c is found in both loops i and j $S_{loop}(i,j)$ be will always be 1. Therefore S_{loop} readily identifies shift permutations. When clusters assigned to W are distinct, S_{loop} is heavily influenced by the similarities between clusters, $S_{cluster}$. This formulation is motivated by the edit distance (Levenshtein distance) for a string in which the similarity of the strings (loops) is calculated by considering the number of character (cluster) replacements, deletions and insertions are required to transform one string into the other. SU sets the cost of each of the potential clusters edits.

We use S_{loop} to cluster the system into a set of loops. The confidence in the estimate of S_{loop} depends strongly on how many times a particular sequence of states is experimentally observed. Thus, we bias the clustering to merge the least traversed loops first. This is accomplished by dividing each entry in S_{loop} by the loop bandwidth:

$$B_{loop}(i) = \sum_{c_j \in W_i} \frac{B(c_j, c_{j+1})}{|W_i|}. \tag{X}$$

Finally, we symmetrize the similarity matrix by:

$$S_{\mathrm{loop}}(i,j) = \max(S_{\mathrm{loop}}(i,j), S_{\mathrm{loop}}(j,i)). \tag{Y}$$

This matrix is then submitted to hierarchical clustering. The output of this clustering is a set of loop clusters C_W. Each C_W is a collection of ordered sequences $W = \{c_i \ldots c_n, c_i\}$ that begin and end in the same cluster of points c. The optimization of this clustering procedure and the assembly of the loop clusters into the final model is described in the next section.

0.9 Assembling Loops into the complete model

To optimize the number of loop clusters, C_W, we use the following heuristic. We would like to choose the number of loop clusters such that the overall model faithfully recapitulates the experimental data. This validation requires two steps. First, an agglomerate average loop has to be constructed from all the loops that together comprise a loop cluster C_W. This operation is done solely on the basis of loops W. In the second step, each phase along W has to be mapped back from the abstract model space onto experimental observations. This last step uses the coordinates of the experimental observations $\mathbf{x}$ that make up each cluster c within W. The number of loop clusters is optimized over a pre-specified range such that this two step process faithfully reconstructs the data. The specifics of how this optimization is accomplished is outlined below.

For each putative number of loop clusters we first need to approximate each C_W by an average loop. This is not trivial, as different loops W are typically shift permutations of each other. Thus, in order to appropriately

average them, the phases of all W within a loop cluster need to be aligned first.

We arbitrarily define 0 to be the most commonly observed cluster, c_{common} amongst all the loops, $W \in C_W$. Now, we define the start for each of each loop as cluster c_{start} such that $S_{cluster}(c_{start}, c_{common})$ is minimized. Because, by definition, each $W$is a loop, given the start state c_{start} the rest of the point clusters c that comprise each W can be assigned a phase, $\theta_{cluster}$ between 0 and 2π using linear interpolation. The phase resolution $\Delta\theta_{cluster}$ therefore is given by the number of states that comprise each W.

What we would like to ultimately achieve is a more continuous definition of phase. To accomplish this, we average locations of data points with respect to the phase of the loop. This is performed using a standard approach consisting of first defining a phase bin with desired granularity and averaging the position of all data points that fall into the phase bin. The procedure for performing this mapping is outlined below.

We first pick the number of phase bins (based on the "total state count" parameter of the method) and construct a Gaussian window centered at each bin. To define the width of this Gaussian window we compute a weighted average of the number of clusters that comprise each loop in C_W. We weigh loop W in C_W in a manner similar to Eq. SX.

$$\Omega_W(i) = \sum_{c_j \in W_i} M_{reduced}(c_j, c_{j+1}) \tag{Z}$$

We can now define the width of the Gaussian phase window as follows:

$$\sigma_{phase} = \frac{\pi}{\sum_{W_i \in C_W} \Omega_W(i) \cdot |W_i|} \tag{AA}$$

where $|\cdot|$ is the number of clusters, c that comprise the i-th loop W. Thus, σ_{phase} is chosen to be approximately 1/2 the average $\Delta\theta_{cluster}$.

Now that we have defined a phase bin, we can interpolate the coarse description of phase given by $\theta_{cluster}$ and identify positions in the data space that are associated with each phase bin.

$$\bar{\mathbf{x}}_{\theta(i)} = \sum_{j \in C_W} exp\left(\frac{-(\theta(i) - \theta_{cluster}(j))^2}{2\sigma^2_{phase}}\right) \cdot \Omega_{cluster}(j) \cdot \bar{\mathbf{x}}_j \tag{AB}$$

where $\bar{\mathbf{x}}_j$ is the average position of all points in cluster c_j, $\theta(i)$ is the i-th phase bin and, finally $\Omega_{cluster}$ is the weight of the cluster defined as follows:

$$\Omega_{cluster}(j) = \sqrt{|c_j|} \cdot \sum_{\{W_k | W_k \in C_W, c_j \in W_k\}} \Omega_W(k) \tag{AC}$$

This equation is scaling the contribution of each point cluster c by its size and by how often it appears in

different loops $W \in C_W$.

The ultimate result of the above is that we approximated each C_W by an average loop. Furthermore, the position along this loop is defined in terms of finely grained phase θ. Finally, each θ is now associated with the average location of the experimental data $\bar{\mathbf{x}}_{\theta(i)}$. Thus, each average loop $\overline{W}$ is now defined as an ordered sequence $\left\{\bar{\mathbf{x}}_{\theta(1)} \ldots \bar{\mathbf{x}}_{\theta(N)}\right\}$ where N is the number of phase bins.

This weighted approximation tends to underestimate the curvature of the loops (Fig SJ). Thus, in order to construct a more accurate model we refine the average loop, $\overline{W}$. First, to make calculation of distances in high dimensional spaces more robust, we perform principal component analysis (PCA) on $\overline{W} = \left\{\bar{\mathbf{x}}_{\theta(1)} \ldots \bar{\mathbf{x}}_{\theta(N)}\right\}$. Both $\left\{\bar{\mathbf{x}}_{\theta(1)} \ldots \bar{\mathbf{x}}_{\theta(N)}\right\}$ and all observed points in the corresponding C_W are then projected onto the first three PCs. For each bin $\bar{\mathbf{x}}_{\theta(i)} \in \overline{W}$, we adjust the bin position to be the mean position of all points in C_W which have this $\bar{\mathbf{x}}_{\theta(i)}$ has their closest bin. This produces an average loop that is able to more closely track the shape of the loops. Thus, each C_W corresponds to a single refined average loop, α.

The result is that each point is assigned a position in model space $\mathcal{M} = \{\theta(i), \alpha\}$. The final step used to simulate model dynamics is to assemble a transition probability matrix M_{model} where each element (i, j) is given by:

$$M_{model}(i, j) = P(\mathcal{M}_t = i | \mathcal{M}_{t+1} = j) \tag{AD}$$

where the conditional probability P is empirically derived from the data and i and j correspond to unique states in model space defined by $\{\theta, \alpha\}$. This transition probability matrix, along with the mapping from state $\{\theta, \alpha\} \mapsto \bar{\mathbf{x}}_i$, makes up the minimal model of dynamics. This model emits a sequence of $\bar{\mathbf{x}}_1, \bar{\mathbf{x}}_2 \ldots \bar{\mathbf{x}}_N$ which can be directly compared to the sequence of experimental observations $\mathbf{x}_1, \mathbf{x}_2 \ldots \mathbf{x}_N$.

The goodness of fit is computed by a validation score of the model. This score approximates the log likelihood of the data given the model. Note that we cannot explicitly calculate the likelihood function because the LOOPER does not explicitly define clusters in terms of probability distributions. Another approach could be to empirically estimate the probability distribution of each cluster from the observed data, at the cost of computational complexity and inaccurate estimates when the number of observations in a cluster is low. For each point in the observed data we compute a measure of how likely it is to observe a specific transition from $\mathbf{x}_t$ to $\mathbf{x}_{t+1}$:

$$score_t = \min_{i \in \mathcal{M}, j \in \mathcal{M}} log(D(\mathbf{x}_t, \bar{\mathbf{x}}_i) \cdot D(\mathbf{x}_{t+1}, \bar{\mathbf{x}}_j))/M_{model}(i, j). \tag{AE}$$

Where $D(\mathbf{x}_t, \bar{\mathbf{x}}_i)$ is the Euclidean distance from the observed point $\mathbf{x}_t$ to $\bar{\mathbf{x}}_i$. We minimize the $score_t$ over all possible combinations of i and j in order to find the best possible combination of states and transition probabilities in M_{model} to describe the experimentally observed data's transition from $\mathbf{x}_t$ to $\mathbf{x}_{t+1}$.

Thus, better scores occur when both $\mathbf{x}_t$ and $\mathbf{x}_{t+1}$ are close to some phase bin $\bar{\mathbf{x}}_i$ of the model, and the transition between those two states, $M_{model}(t, t+1)$, is highly probable. The total validation score is the mean score over the entire time series of experimental observations.

We optimize the number of loops by fixing the number of states in $\mathcal{M}$ (the "total state count" parameter of the method). We iterate this validation procedure for each putative number of clusters, C_W, and choose the final number of clusters such that the total validation score is minimized. This concludes the process that is used by LOOPER in order to deconstruct the dynamics into a collection of interconnected loops.

0.10 Simulating LOOPER model with inputs

In order to construct a complete LOOPER model of a recurrent neural network we augment the unit activity of the RNN with both its inputs and outputs. This results in a $n+2 \times t$ matrix where n is the number of hidden variables, and t the number of timesteps. Without inputs, simulation of the LOOPER model is identical to simulation of a Markov matrix. At each time step the current state of the simulation transitions to a new state with probability equal to the corresponding row of the matrix, M. With the addition of inputs, we must consider not only the current state of the system, but also the current input to the system. This is done by weighting the transition probabilities of the current state by the input value:

$$w_i = exp\left(\frac{-(I(t) - \bar{i_i})^2}{2\sigma_i^2}\right), \tag{AF}$$

where i is the next state ID, $I(t)$ is the current input, $\bar{i_i}$ is the mean input value of state i and σ_i is the standard deviation of the input at state i. This means that the system at state j will be more likely to jump to the next state i when the input is similar to the mean input of state i. This value is clipped to 0 if less than $exp(-2)$. The final probability of jumping from state j to state i is $M_{ji} * w_i$. However, due to the precise temporal timings of inputs in the task, inputs are sometimes missed. This can occur when the simulated system traverses too quickly and randomly jumps out of a state that should only be transitioned out of during inputs. In order to help alleviate this issue we allow some leeway in the timing of inputs.

If the inputs into the system are such that no valid transitions exist from the current state (i.e. all transition probabilities are less than $exp(-2)$), then we draw from the previous state of the simulation instead. This can repeat several times so that the simulation can look several time steps into the past for the most recent state for which a valid input exists. For the simulations in Fig 3C backtracking occurs on only 5% of time steps and the mean backtracking time is 3.7 time steps (max 12). Thus, this method still produces a good approximation of the true time evolution of the system

0.11 Extending LOOPER to work with trial data

LOOPER's loop clustering algorithm specifically assumes that the data forms complete loops in state space. This is true in continuous records such as pan-neuronal calcium imaging in *C. elegans*, but in short trial-based recordings this may not be the case. Note that in reality all neuronal data is continuous, but experimental design may be such that parts of the recordings are left out. In order to maintain the assumption of continuity in the data we manually add a single hidden state to the LOOPER model. This hidden state represents all configurations of the system that are not observed in the recorded data, but none-the-less present in the actual neuronal activity. Thus, this state cannot be transitioned to in the observed data. Instead, we assume that every trial transitions to this state after the last observed time point, and that the first time point of a trial transitions from this hidden state. In this way, each trial can be considered a closed loop beginning and ending in the hidden state. Note that the inclusion of this state does not add any information to the dataset as it is condition agnostic.

0.12 State space extraction

Before dynamics can be appropriately simulated, the state space – a set of salient variables that influence the system dynamics – must be extracted from the experimental observations. The procedure used to extract state space from experimental observations is described in this section. When working with a complete set of latent variables (state space) there is a one to one mapping between the position of the system, $\mathbf{x}$ and the flux of the system $\mathbf{F}(\mathbf{x})$. Commonly, the latent variables that make up the state space are missing or hidden. These missing latent variables can cause a degeneracy in the equations of motion of the system where the same $\mathbf{x}$ gives different values of $\mathbf{F}(\mathbf{x})$. When this occurs, information about the coherent trajectories in the system is lost and LOOPER will fail to find the corresponding loops – it may instead find a stochastically dominated version of the loops merged together. Thus, an important preprocessing step is to make sure that the data contains all the information required to find the latent variables.

There are many techniques for latent variable extraction such as LFADS [6], Hidden Markov Models [7], MIND [8] and NNMF [9], etc, and any can be used in preprocessing before applying LOOPER to the data. LOOPER itself has delay embedding built in. Delay embedding is a method for state space extraction that relies on increasing the number of independent measurements from experimental observations in order to extract a topologically equivalent representation of the underlying dynamics. To extract these independent measurements, a delay time τ is chosen such that the autocorrelation between point $\mathbf{x}_t$ and $\mathbf{x}_{t+\tau}$ is small. Several of these time delayed observations are added to each observed time point in the data. There are many possible techniques for choosing the ideal number of delays and the correct value of τ. LOOPER leaves these values as free parameters.

Generally, one can over embed and rely on LOOPER's dimensionality reduction to remove excess dimensions. Takens' theorem guarantees that the delay embedded dynamics will be topologically equivalent to the underlying dynamics, thus implying that the loop structure will not change.

0.13 Parameter Exploration

The LOOPER model depends critically on 3 parameters. Number of nearest neighbors used in diffusion map construction, the transition probability repopulation density, and the target state count for construction of the final model. The parameters used for the figures in the main text are shown in Table 2. We explore the sensitivity of the method to these parameters choices by rerunning LOOPER on the primate data with different parameter values. We then compare the extracted scaffold with new parameter values to the "ideal" scaffold which separates conditions based on F1 before the F2 stimulus, and after the F2 stimulus separates all conditions. The decoding rate is given by the sum of the difference in decoding rates of the scaffold over these time periods (50% for F1, and 100% for F2). The performance of the method as a function of different parameter choices is shown in Fig SH. Only the number of nearest neighbors makes an appreciable impact on the quality of the model. Note that the accuracy falls off sharply after increasing past the number of trials per condition (10). Most of the deficiencies of the reconstruction are limited to the incorrect estimation of the time at which loops branch. Over a broad range of parameters, the reconstructed branch structure remains unchanged.

0.14 Hidden Markov Model Fitting

Hidden Markov models were fit using the EM algorithm as implemented by the BNT toolbox. We fit the time series data after state space discovery in order to ensure that both LOOPER and HMMs are trained on the same inputs. We further z-scored all channels of the data and fixed the covariance matrices of the hidden states to either 0.1 or 0.2 along the diagonals with no off-diagonal elements (See Table 3 for full list of hyper-parameters). In order to keep HMMs as competitive as possible we apply standard diffusion mapping to the data before running HMM. This gives 5 total hyper-parameters for HMM fitting: epsilon (size of sigma in diffusion kernel), t (number of times to exponentiate the diffusion map), k (number of modes of the diffusion map to keep), PC count and the number of states of the system. Note that LOOPER's 3 parameters correspond to these hyper-parameters: number of nearest neighbors → epsilon, repopulation density → t, and target number of states → state count. Hyper-parameters were then optimized using Bayesian Optimization (See Table 3 for full list of optimal hyper-parameters).

0.15 Preprocessing of primate data

Preprocessing of the primate data is done using the same pipeline as in Kobak et al [10]. Spike rasters are converted to firing frequencies by using a 30ms Gaussian sliding window. Only trials with average firing frequencies between 5Hz and 50Hz are considered. We restrict the analysis to neurons observed on at least 10

trials in each condition (179 neurons over 6 trial conditions total).

Neurons are recorded in separate sessions. Thus, we bootstrap over trials in order to create randomized pseudotrials which consist of time synchronized firing of all neurons using the stimulus times (Fig 5B). Each pseudotrial is constructed by taking the mean firing of each neuron during a given condition averaged over $n/2$ bootstraps randomized with replacement. Here, n is the number of trials for that particular neuron and condition. For Fig 4B the model is trained on trials with odd trial indices, and validated on trials with even indices (Fig SD).

0.16 *C. elegans* data

We validate the LOOPER model on Ca2+ imaging data published by Kato et al. [11]. The dataset consists of 5 individual C. elegans immobilized in a microfluidic device [12] under environmentally constant conditions. Each recording contains between 107 to 131 neurons spanning the head ganglia and head motor neurons, along with most sensory neurons and interneurons of the anterior ventral cord motor [13]. In order to build a dataset that spanned all 5 animals, we restricted our time series to the subset of 15 neurons that were unambiguously identified in each animal (AIBL, AIBR, ALA, AVAL, AVAR, AVBL, AVER, RID, RIML, RIMR, RMED, RMEL, RMER, VB01, VB02). Neuronal activity is measured by the deviation of fluorescence from baseline ($\Delta F/F$). Preprocessing was applied by the Zimmer Lab to account for photobleaching of the GCaMP during the ~15 minute recordings.

0.17 Mouse data

Animals: All experiments in this study were approved by Institutional Animal Care and Use Committee at the University of Pennsylvania and were conducted in accordance with the National Institutes of Health guidelines. All experiments were performed using adult (12-32 weeks old, 20-30g) male and female C57BL/6 mice (Jackson Laboratories). Mice were housed under a reverse 12:12 h, light: dark cycle and provided food and water *ad libitum*. We present data from 5 mice in this study.

Headplate implantation and habituation: At least 2 weeks before recording, mice were chronically implanted with custom designed headpieces for head-fixation during awake recordings. Under isoflurane anesthesia (1.5% isoflurane in oxygen), the animal was secured in a stereotaxic frame (Narishige). 8 mg/kg bupivacaine was injected in the temporal and neck muscles. Betadine was applied to sterilize the exposed skin. After scalp was resected, bregma and lambda were marked. Custom made headpiece was attached using dental cement (Metabond) and 3 skull screws. Loctite 495 was applied over any remaining exposed skull. Mice were given 5 mg/kg mg/kg meloxicam, 10 mg/kg of carparfen, and 7 ml of normal saline SQ after surgery and left to recover

for a week before starting the habituation protocol. Mice were habituated to head fixation with body restraint with visual stimuli gradually over the course of 4 days. By the end of day 4, mice tolerated awake head fixation and visual stimuli for 45 minutes uninterrupted.

Craniotomy: After habituation, animals were anesthetized with isoflurane and secured in the stereotaxic frame using the headpeace. Before surgery, subctaneous (8 mg/kg bupivacaine) and systemic analgesia (5 mg/kg mg/kg meloxicam, 10 mg/kg of carparfen) were administered. 2 mg/kg dexamethasone was administered to prevent brain swelling. Left sided craniotomy (+1mm to -5 mm AP, +0.25 mm to +6 mm ML of bregma) was performed using standard technique to expose dura. A 64-electrode surface grid (Neuronexus: E64-500-20-60) was positioned over the dura (anterior and midline most electrode 1 mm lateral and 1 mm posterior to bregma). The grid and exposed dura was then covered with gel foam soaked in mineral oil to protect the brain from desiccation and achieve maximum electrode contact.

Visual Stimulation: Recordings in the awake state began 30 minutes after isoflurane discontinuation. Hundred visual stimuli (10 ms flashes of a green LED light; 650 cd/m2, placed 2 cm away from the mouse's right eye) covering 100% of the mouse's visual field were administered and their delivery synchronized to the electrophysiological recordings

Electrophysiology and preprocessing: Electrophysiological signals were amplified via an Intan headstage (Intan, RHD2132), digitized through Omniplex acquisition system (Plexon, Omniplex), and collected at a rate of 30,000 samples/second. LFP data was filtered offline using a custom-built FIR filter between 0.1 Hz and 325 Hz, with the MATLAB functions, firls.m and filtfilt.m. Offline, data sets were decimated to 1000 samples/second, noise channels were manually removed and trials with excess motion artifact were manually rejected. All LFP data were re-referenced to the mean to decrease the common ground artifact.

Analysis: We first bootstrap the data by averaging the activity of each electrode over a randomized subset of 5 single trials. 50 such bootstrapped trials were constructed and projected onto the top 8 PCs (95% variance explained of all raw trials). These time series were then downsampled to 100Hz, and submitted to LOOPER (See Table 2 for full list of parameters used). We found that the results presented are robust to changes in parameters.

0.18 fMRI data

Data source and participants: We obtained permission to use the Open HCP dataset. For the current analysis, we employed the 1200 Subjects Release (S1200) data set, which included behavioral and 3T MR imaging data of 1029 healthy young adult participants. Four participants were excluded due to missing fMRI data. An additional 254 participants were excluded because they responded "I don't know" in the theory of mind (see details below),

resulting in 771 participants included in the current dataset. Participants were between 22 and 36 years old (22-25yo: 20.8%; 26-30: 43.5%; 31-35: 34.9%; 35+: 0.8%), and 55.8% female. All subjects were healthy adults with no severe neuropsychiatric disorders. Each participant provided informed written consent (including consent to share de-identified data), and this study was approved by the Institutional Review Board at the University of Washington.

Theory of Mind task: During the task (Fig 4A), participants were shown video clips in which geometric shapes (triangles, squares, and circles) either "interacted" in some way to simulate social interactions ("social condition"), or moved randomly ("random condition") [14, 15]. Each participant completed two runs of the task, and each run included five blocks. In each block, the participants were exposed to a 20s movie clip (adapted from the original 40s version [16]). At the end of each video clip, participants were asked to respond whether the objects interact socially (as if the objects were aware of each other's thoughts and feelings), if they moved randomly, or not sure. Participants were allowed 3s to respond. For the current analysis, we focused the most ambiguous movie (block #9, "random mechanical"), and limited our analysis to the participants that responded either "social interaction" or "random movement".

fMRI image acquisition and preprocessing.. Participants were scanned on a customized Siemens 3T "Connectome Skyra" housed at Washington University in St. Louis. The customized scanner used a standard 32-channel head coil and a body transmission coil. T1-weighted high-resolution structural images were acquired using a 3D MPRAGE sequence (isotropic resolution: 0.7 mm, FOV = 224 x 224 mm, matrix = 320 x 320, 256 sagittal slices, TR = 2400 ms, TE = 2.14 ms, TI = 1000 ms, FA = 8°). The T1-weighted structural images were used to register functional MRI data to a standard brain space. Functional MRI data were collected using gradient-echo echo-planar imaging (EPI; isotropic resolution:2.0 mm, FOV = 208 x 180 mm, matrix = 104 x 90, 72 slices, TR = 720 ms, TE = 33.1 ms, FA = 52°, multi-band factor = 8, 274 frames, ˜3 m and 27 s/run). Preprocessing was done using the HCP MRI data preprocessing pipelines [17]. The brain regions of interest (ROIs) were defined based on a prior study that utilized functional connectivity-based method to generate an atlas that separated ROIs into distinct functional networks [18]. This atlas includes 264 ROIs in both cortical and subcortical spaces. The preprocessed data were then bandpass filtered between 0.06-0.45 Hz [19], detrended, standardized, and extracted from 5-mm-radius spheres around the ROIs . Artifacts were reduced using nuisance regressors including white matter and ventricular signals, the realignment parameters, and high-variance confounds as implemented in the nilearn package [20].

Analysis: We first bootstrapped the data by averaging the activity of each parcel over a randomized subset of 60 single trials for each condition (responded "Yes" and reponsed "No"). Trials in which the subject responded "Don't know" were ignored. 10 bootstrapped trials were constructed for each condition, starting 15 frames

(~10.8s before the movie, and ending 7.2s after the movie). All 264 parcels were submitted to LOOPER (See Table 2 for full list of parameters used). In order to ensure robustness we repeated the LOOPER analysis over a range of parameters with 5 bootstrap randomizations per parameter set.

In order to find the parcels with the highest separation we consider the time period in which the trajectories of the two conditions separate most reliably (Fig 4C). We calculate the d' (sensitivity index) of each parcel for each time point and take the maximum d' during this time period of separation. This process is repeated for 100 randomizations of the bootstrapping procedure, and a representative sensitivity index is given by the median value of the distribution of maximum d's over the time period of separation. The 20 parcels with the highest d' are plotted in Fig 4D.

0.19 Training of Recurrent Neural Networks

The RNNs in Fig 4C and Fig 3 consist of 100 fully connected LSTM units, and were trained with PyTorch using Adam with 10000 randomized samples. Inputs were a time series of scalar values (0, F1 or F2) corrupted by Gaussian noise with a sigma of 1.5. The loss function was standard cross entropy on the 3 output classes ("No output", "Greater than", and "Less than") weighted so that the target outputs ("Greater than" or "Less than") contribute the same amount as the "No output" cases. This means that the network must match the given target of "No output" for all time points other than the short target window directly after F2. During this window it is heavily penalized for missing the target output. The models are trained until they have a loss of less than 0.01. Classification accuracy of the network is assessed by taking the mode of the outputs during the target window directly following F2.

In order to increase the biological realism of the network we inject Gaussian noise with a sigma of 0.1 into both the hidden and cell state of the LSTM units at each time step. The values of the hidden and cell states have a standard deviation of 2.3 over all observations, so the noise is roughly 4% of the signal.

References

1. Teschl G. Ordinary differential equations and Dynamical Systems. vol. 140. American Mathematical Soc.; 2004.

2. Nadler B, Lafon SS, Coifman RR, Kevrekidis IG, Kevrekedis IG. Diffusion maps, spectral clustering and reaction coordinates of dynamical systems. Applied and Computational Harmonic Analysis. 2006;21(1):113–127. doi:10.1016/j.acha.2005.07.004.

3. Wang J, Xu LL, Wang E. Potential landscape and flux framework of nonequilibrium networks: Robustness, dissipation, and coherence of biochemical oscillations. Proceedings of the National Academy of Sciences. 2008;105(34):12271–12276. doi:10.1073/pnas.0800579105.

4. Beretta A, Battistin C, de Mulatier C, Mastromatteo I, Marsili M. The stochastic complexity of spin models: Are pairwise models really simple? Entropy. 2018;20(10):739. doi:10.3390/e20100789.

5. Dijkstra EW. A note on two problems in connexion with graphs. Numerische Mathematik. 1959;1(1):269–271. doi:10.1007/BF01386390.

6. Pandarinath C, O'Shea DJ, Collins J, Jozefowicz R, Stavisky SD, Kao JC, et al. Inferring single-trial neural population dynamics using sequential auto-encoders. Nature Methods. 2018;15(10):805–815. doi:10.1038/s41592-018-0109-9.

7. Vilares I, Kording K. Bayesian models: The structure of the world, uncertainty, behavior, and the brain. Annals of the New York Academy of Sciences. 2011;1224(1):22–39. doi:10.1111/j.1749-6632.2011.05965.x.

8. Low RJ, Lewallen S, Aronov D, Nevers R, Tank DW. Probing variability in a cognitive map using manifold inference from neural dynamics. bioRxiv. 2018; p. 418939. doi:10.1101/418939.

9. Anderson A, Douglas PK, Kerr WT, Haynes VS, Yuille AL, Xie J, et al. Non-negative matrix factorization of multimodal MRI, fMRI and phenotypic data reveals differential changes in default mode subnetworks in ADHD. NeuroImage. 2014;102(P1):207–219. doi:10.1016/j.neuroimage.2013.12.015.

10. Kobak D, Brendel W, Constantinidis C, Feierstein CE, Kepecs A, Mainen ZF, et al. Demixed principal component analysis of neural population data. eLife. 2016;5(APRIL2016):e10989. doi:10.7554/eLife.10989.

11. Kato S, Kaplan HS, Schrödel T, Skora S, Lindsay TH, Yemini E, et al. Global Brain Dynamics Embed the Motor Command Sequence of Caenorhabditis elegans. Cell. 2015;163(3):656–669. doi:10.1016/j.cell.2015.09.034.

12. Schrödel T, Prevedel R, Aumayr K, Zimmer M, Vaziri A. Brain-wide 3D imaging of neuronal activity in Caenorhabditis elegans with sculpted light. Nature methods. 2013;10(10):1013–1020. doi:10.1038/nmeth.2637.

13. White JG, Southgate E, Thomson JN, Brenner S. The structure of the nervous system of the nematode Caenorhabditis elegans: the mind of a worm. Phil Trans R Soc Lond. 1986;314(1165):1–340. doi:10.1098/rstb.1986.0056.

14. Castelli F, Happé F, Frith U, Frith C. Movement and mind: a functional imaging study of perception and interpretation of complex intentional movement patterns. Neuroimage. 2000;12(3):314–325.

15. Wheatley T, Milleville SC, Martin A. Understanding animate agents: distinct roles for the social network and mirror system. Psychological science. 2007;18(6):469–474.

16. Barch DM, Burgess GC, Harms MP, Petersen SE, Schlaggar BL, Corbetta M, et al. Function in the human connectome: task-fMRI and individual differences in behavior. Neuroimage. 2013;80:169–189.

17. Glasser MF, Sotiropoulos SN, Wilson JA, Coalson TS, Fischl B, Andersson JL, et al. The minimal preprocessing pipelines for the Human Connectome Project. Neuroimage. 2013;80:105–124.

18. Power JD, Cohen AL, Nelson SM, Wig GS, Barnes KA, Church JA, et al. Functional Network Organization of the Human Brain. Neuron. 2011;72(4):665–678. doi:10.1016/j.neuron.2011.09.006.

19. Shirer WR, Jiang H, Price CM, Ng B, Greicius MD. Optimization of rs-fMRI Pre-processing for Enhanced Signal-Noise Separation, Test-Retest Reliability, and Group Discrimination. NeuroImage. 2015;117:67–79. doi:10.1016/j.neuroimage.2015.05.015.

20. Schmälzle R, O'Donnell MB, Garcia JO, Cascio CN, Bayer J, Bassett DS, et al. Brain connectivity dynamics during social interaction reflect social network structure. Proceedings of the National Academy of Sciences of the United States of America. 2017;114(20):5153–5158. doi:10.1073/pnas.1616130114.

21. Golub M, Sussillo D. FixedPointFinder: A Tensorflow toolbox for identifying and characterizing fixed points in recurrent neural networks. Journal of Open Source Software. 2018;3(31):1003. doi:10.21105/joss.01003.

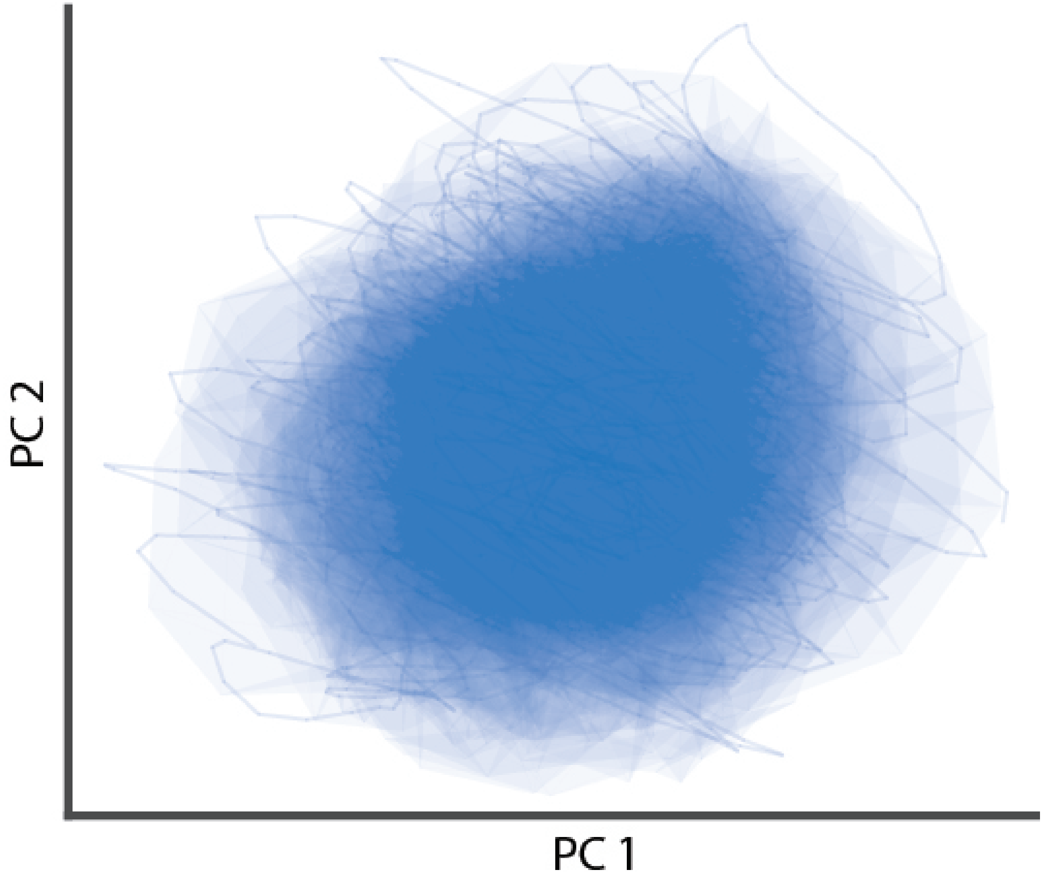

Fig A. LOOPER Model of Untrained RNN. An untrained RNN perturbed only by Guassian noise. The dynamical structure is a single fixed point with small deviations due to the noise (thin blue lines are traces). The data was subjected to the same preprocessing as the RNN data used in the paper. The shaded blue regions represent the bins found by LOOPER (same as Fig 1E). Note that LOOPER finds only the single fixed point – and no loops – when run on this system.

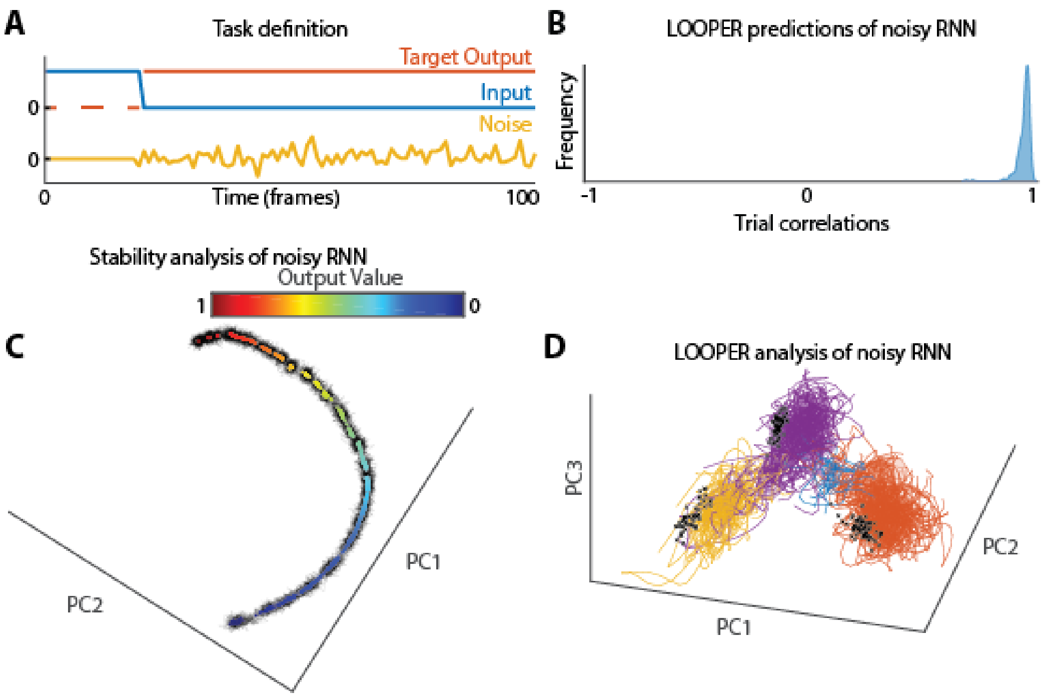

Fig B. **Linear Stability Analysis and LOOPER. A)** The network gets an input value between 0 and 1 for 20 frames, and must remember this value over the next 80 frames (Blue line). The target output is ignored for the input frames (dotted orange line). Network noise is added after the first 20 frames (yellow line). The network is trained in the same manner as networks in the rest of the paper (See **Methods: Training of Recurrent Neural Networks**). **B)** A histogram of the correlations values between the activity of a network trained to remember an input value, and LOOPER model predictions (same as in Fig 5F-I). **C)** Fixed points in noisy LSTM dynamics are found by minimizing the amount of flux for each putative fixed point. The procedure follows code provided by Sussillo et al. [21]. The fixed points are colored by their projection onto the output value, and projected onto the first 3 PCs of the hidden states of the network. **D)** Trajectories of the system colored by assigned LOOPER trajectory ID when running on a subset of input values. Black crosses denote the locations of the fixed found using stability analysis. Note that the clustering preformed by LOOPER is in good agreement with the structure of the fixed points found by the methods of Sussillo et al. The blue cluster corresponds to a few trajectories that cross between the purple and orange clusters, and cannot be easily assigned to one or the other - this cluster has very low occupancy and can be safely ignored.

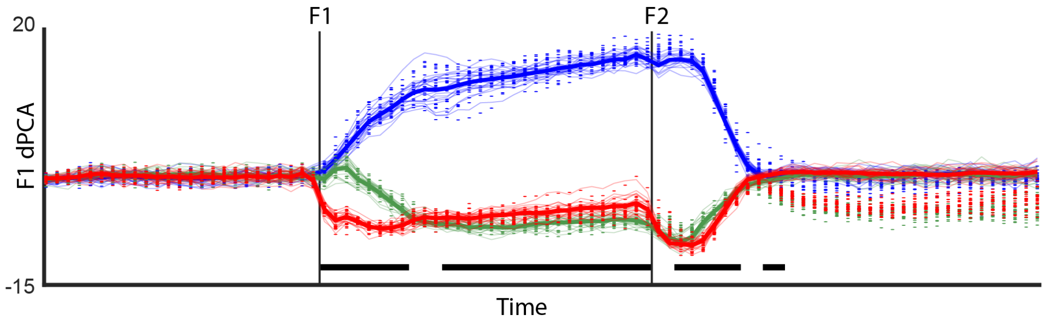

Fig C. **Supervised learning techniques extract erroneous scaffold.** RNN activity (Fig 5) projected onto the dPCA which maximizes differences in the F1 condition (F1=10 in blue, F1=20 in green and F1=40 in red). At each time point, the Kruskal-Wallis one-way Anova test is used to calculate a probability that the data from the F1 = 20 (green points) and F1 = 40 (red points) are drawn from the same distribution. The thick black line at the bottom shows all time points in which this test is statistically significant ($p < 0.01$). Note that the two F1 conditions are statistically different at the onset of F2. Empirical testing using novel stimuli (main text), however, shows that the response of the system at this point is the same regardless of whether F1=20 or F1=40.

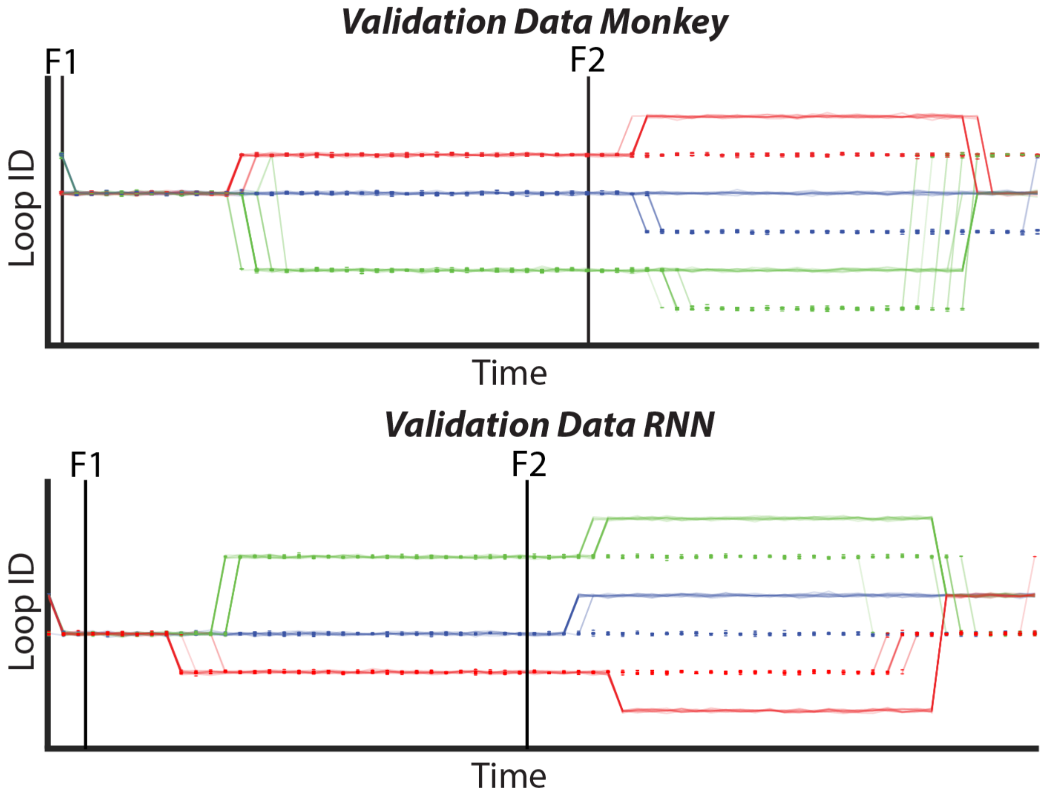

Fig D. Validation of Computational Scaffolds. Validation of LOOPER computational scaffold formed by projecting the left out data from the monkey and a unique set of data from the RNN onto the original computational scaffold as described in the Methods section. Primate validation accuracies and p-values are listed in the main text. RNN accuracies are: classifying F1 $\rightarrow$ accuracy 94%, p-value < 0.0001, remembering F1 $\rightarrow$ validation accuracy 86%, p-value < 0.0001, comparing F2 to the remembered F1 $\rightarrow$ validation accuracy 98%, p-value < 0.0001, and over all accuracy 95%, p-value < 0.0001.

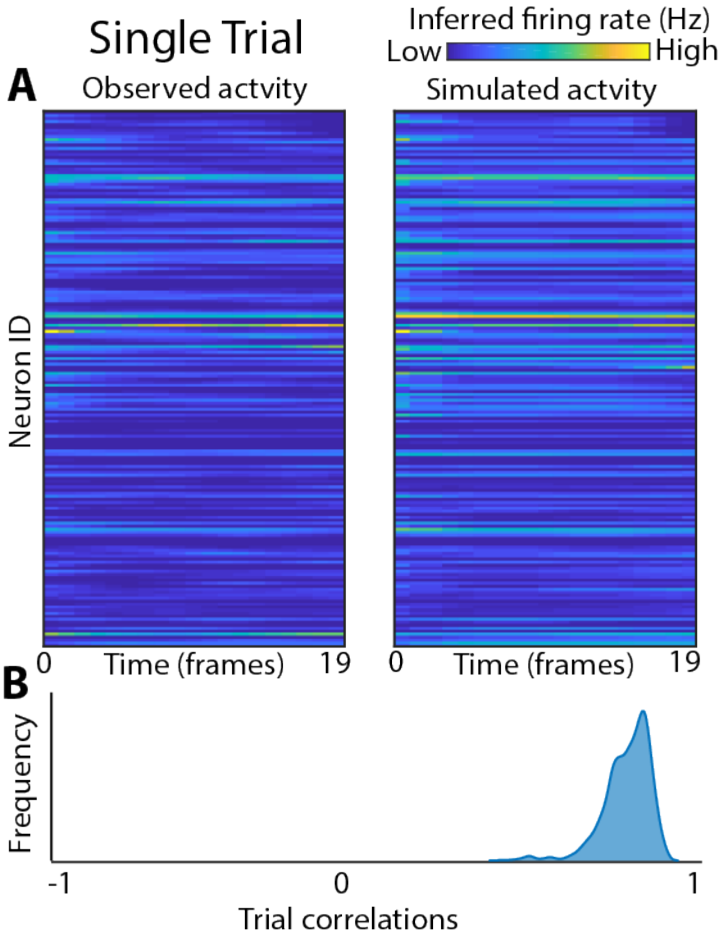

Fig E. LOOPER Model Predicts Neuronal Activity of Single Trials. Same as Fig 5C, but predicting single trials instead of bootstrapped trials. Single trial predictions are made using the same LOOPER model as in Fig 5. Instantaneous firing rate is estimated using a 200ms Gaussian window, up from 30ms in the main text. **A)** (Left) Observed firing rates of monkey prefrontal cortex neurons during a single trial of the working memory task. (Right) Simulated neuronal activity from the LOOPER model over 19 frames (∼1.5s). See Fig 5 for methods. **B)** Distribution of correlation coefficients computed across all trials. See Fig 5 for methods.

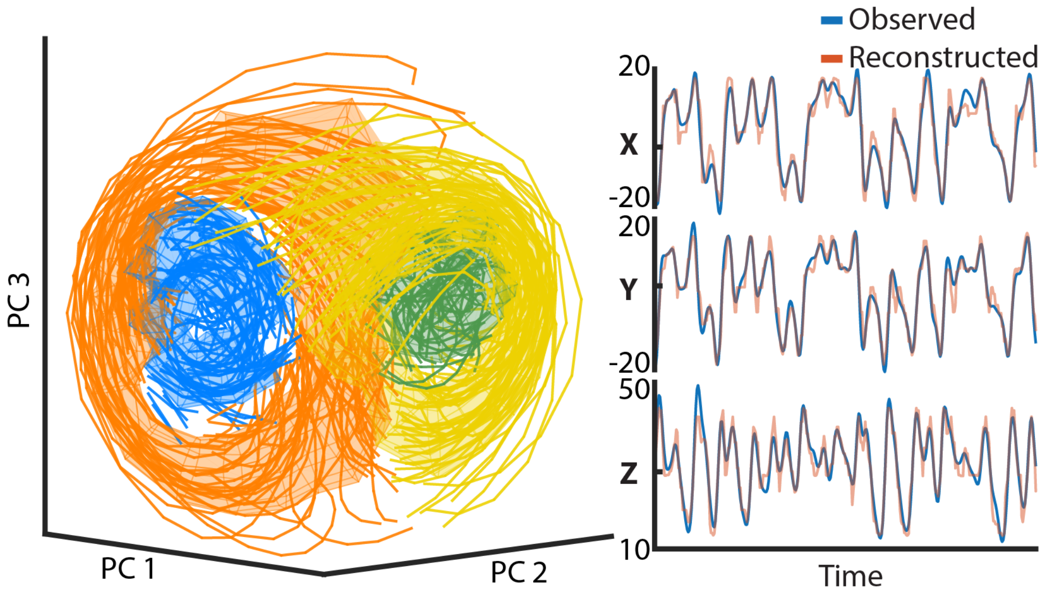

Fig F. LOOPER Model of Lorenz system. Left) Simulation of Lorenz system with Gaussian noise (sigma=1) added at each time-step. Colored by extracted loops. **Right)** LOOPER reconstruction of the Lorenz system. Mean across-variable correlation is 0.96.

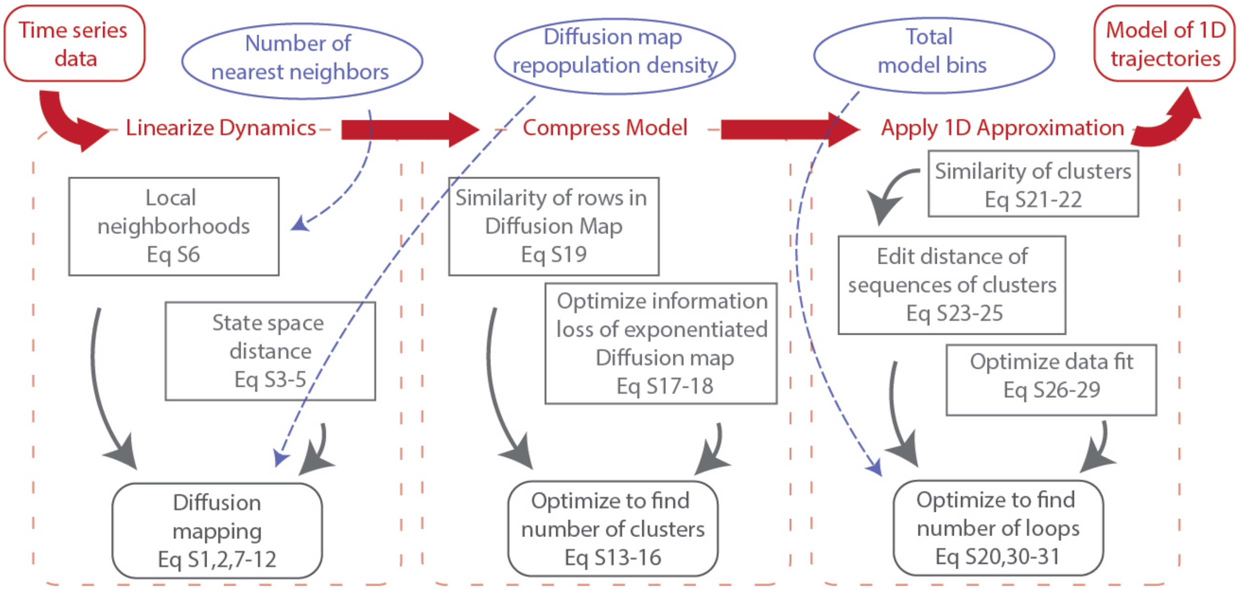

Fig G. LOOPER flowchart. LOOPER takes complex nonlinear time series data and outputs a simple model consisting of interlocking one dimensional trajectories. LOOPER consists of 3 primary sections: Linearizing the dynamics, compressing the model and applying the one dimensional approximation (Red arrows). Details about each section can be found in the supplement. The pertinent equations are highlighted. LOOPER requires 3 parameters (blue arrows), of which only the number of nearest neighbors have an appreciable effect on the output of the model.

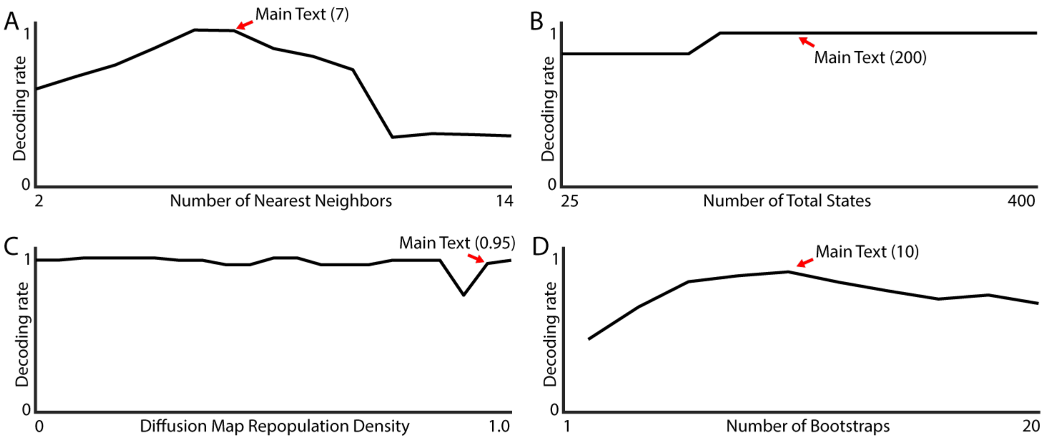

Fig H. **Range of LOOPER parameters.** Range of the three LOOPER parameters – number nearest neighbors (**A**), total number of states in the model (**B**), and the repopulation density of the diffusion map (**C**). We retrained the LOOPER model on these parameter ranges and quantified the goodness of each model by the probability of being able to decode the condition from which the given datapoint came. Note that during the interstimulus period this means decoding the F1 value of the condition, as information about F2 is not expected to be present at that time. These values are averaged over the appropriate time periods (inter-stimulus interval for F1 and after F2 for comparison). **A)** The effect of the number of nearest neighbors on the overall decoding rate of the LOOPER model. Red arrow is the number of delays used in the main text (Fig 2 and Fig 3). The loss of decoding rate at low numbers of nearest neighbors occurs due to the model erroneously splitting trajectories into unique conditions before the F2 stimulus is observed. After 10 nearest neighbors the decoding rate begins to fall off sharply. This is because the data set has only 10 trials in each condition, so using more nearest neighbors causes cross condition merging. **B)** Same as **A** for total states in the model. There is little to no impact of number of states on the decoding power of the model. For all models, even if the decoding rate begins to decay, the overall structure of the models' computational scaffold is not greatly effected – only the timings of the branches. Thus, the computational scaffold extracted by LOOPER is largely robust to changes in parameters. **C)** Same as **A** for repopulation density of the diffusion map. There is little to no impact of repopulation density on the decoding power of the model. **D)** While not part of the LOOPER methodology itself, we also explored the effect of number of bootstraps used on the resultant models. Around 6 to 12 bootstraps are required to build an accurate model of the dynamics.

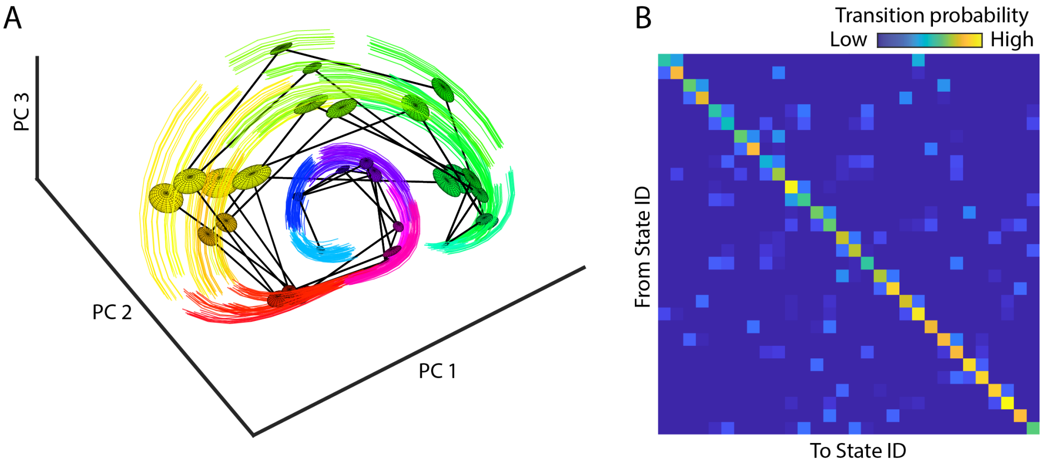

Fig I. Reduced Diffusion Map of the Primate Data. A) Mean and standard deviation of point clusters are shown as the center and standard deviation of each ball respectively projected onto the first 3 principal components. Lines denote transitions between clusters with more than 10% probability. Clusters are colored by mean time since trial onset. The points corresponding to each cluster are plotted as trajectories with the same color as the ball. **B)** Reduced transition probability matrix. Each element is the probability that a point in the given cluster will transition to a point in another cluster. Experimental observations that comprised parallel diagonal bands in Fig 1C are combined into a single cluster in the reduced transition probability matrix. Data is the same primate data as shown in the main text.

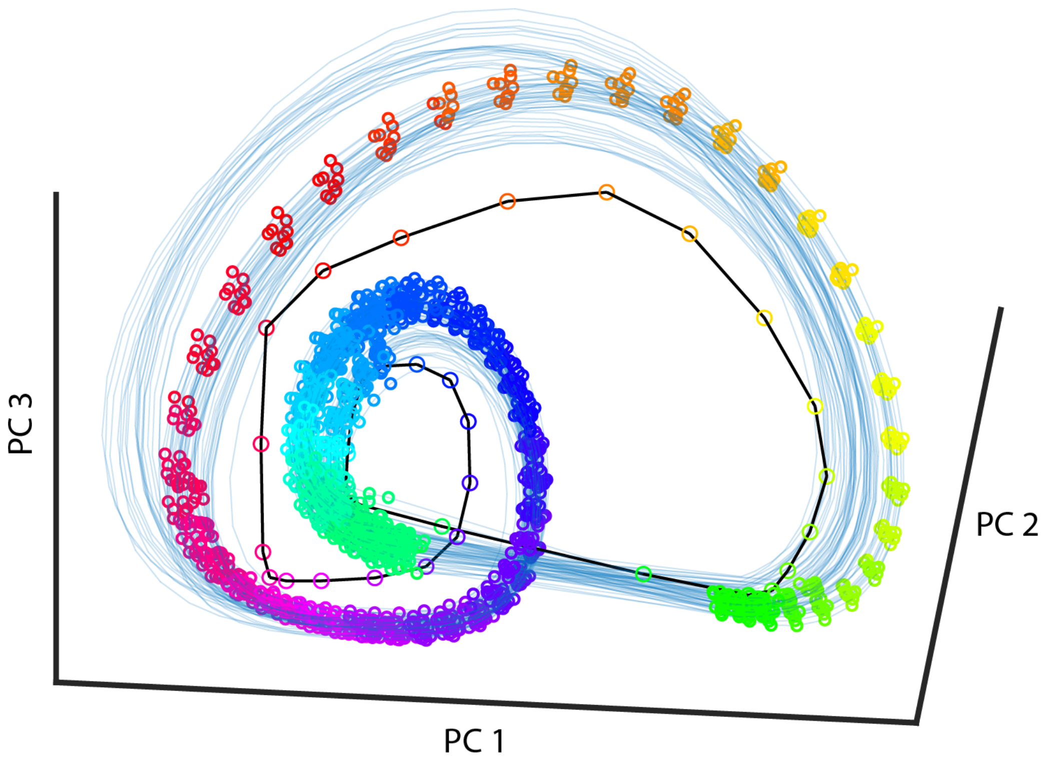

Fig J. **Loop Clustering of Primate Data.** The black line denotes the first approximation of the loop. Each circle on this line denotes a bin, $\bar{\mathbf{x}}_{\theta(i)}$, colored by its associated phase. Note that this Gaussian smoothed loop consistently underestimates the curvature of the data. To form the final loop, points are assigned a phase value based on proximity to the approximate bins (colored points). Position of these points is then used to refine the shape of the final loop. Only points belonging to this loop are plotted for visual clarity. Data is the same primate data as shown in the main text.
