# Supplementary material for: One dimensional approximations of neuronal dynamics reveal computational strategy
Source: PLoS Comput Biol. 2023 Jan 6;19(1):e1010784. doi: 10.1371/journal.pcbi.1010784 (PMC9821456; doi:10.1371/journal.pcbi.1010784)
Supplement: Attachment — Submitted filename: Response to reviewers.pdf [file pcbi.1010784.s002.pdf]

Rebuttal:
We wanted to thank the reviewers for their insightful critiques of our manuscript. Below we addressed every specific concern raised by the reviewers. As a result, we believe that the manuscript is much improved. Thank you.

Reviewer #1: This paper develops a method ("LOOPER") for extracting the core features of a neural population trajectory into a kind of "scaffolding" that can then be interpreted and used to compare the computational strategies used by very different systems. The method proceeds by constructing normalized diffusion maps from neuronal population time series data. This serves as a Markov model of the observed dynamics, whose states are then clustered and continuous trajectories are extracted. This method is successfully demonstrated on several interesting examples from a variety of systems.

I found the method and the results obtained to be quite interesting. The method is well-described and the results obtained across a diversity of systems were impressive. Thus, I support the publication of this paper with just a few minor comments for the authors to consider before finalizing their paper.

Thank you

1) The terminology of "1-dimensional trajectories" used throughout the paper seems odd to me. Aren't all trajectories 1D objects?

We agree that the term is a bit confusing. We wanted to differentiate the case in which a trajectory is stable (all dimensions orthogonal to the flow of the trajectory are robust to noise) and cases in which the trajectories form 2+ dimensional sheets (which can occur if trajectories are aligned along marginally stable dimensions). A sheet of trajectories would be observed in continuous attractor systems (e.g. line attractor). In contrast, our 1-dimensional trajectories are akin to a discrete approximation of a line attractor. We have added a sentence when we introduce stable trajectories to clarify this point.

Line 45
“Note that such trajectories will inevitably be one-dimensional, and cannot be combined to form higher dimensional structures (such as sheets) as they do not contain marginally stable directions orthogonal to the flow.”

2) What exactly does a "stable trajectory" mean (e.g., line 146 and elsewhere)? Stable w.r.t. what, exactly? Perturbations to the neuronal populations? Or multiple runs of LOOPER with different meta-parameter settings? Or something else?

Thank you for raising this important point. Sorry for omitting it from the manuscript. By stable we mean stable with respect to perturbations. Specifically, we expect there to be only one non-negative derivative, and the eigenvalue of that derivative will be in the direction of the flow. We have added a sentence when we introduce stable trajectories to clarify this point.

Line 43
“Stable trajectories occur when all directions in the local neighborhood orthogonal to the flow of the trajectory push the state of the system back to the trajectory, making it robust against local perturbations due to noise”.

3) I wonder if the authors could comment a bit more about the sensitivity of this method of analysis to the scale of the coarse-graining applied? It would seem that the particular scaffolding structure extracted by LOOPER could be heavily dependent on this scale.

This is a very important point. Thank you. There are two parameters in the model that define the scale of the coarse graining. The first is the number of nearest neighbors used to build the diffusion map. We explored the effect of the number of nearest neighbors in Supplementary Figure S8A. The results are most heavily affected when the number of nearest neighbors is larger than the total number of similar trials in the dataset. The diffusion map then incorrectly attempts to merge together different conditions into the same stable trajectory. The second step of the coarse graining is controlled by an optimization that attempts to create a minimum description length model of the original diffusion map, and so is not controlled by a parameter. However, we agree that it is not a priori obvious that this is the optimal cost function to use. The exact state count in the reduced diffusion map model doesn't seem to affect model performance heavily. If the number of states is increased, the final step of finding loops will still stitch the states together into the same (or at least similar) loop. This is illustrated in Supplementary Figure S9, where each state in the reduced diffusion map model is shown by the colored balls. Each state is made up of several trajectories that last for a few time points, forming arcs along the stable trajectories of the data. If the number of states is increased then the number of time points in these arcs will change, but the clustering of trajectories will remain.

4) Although the extracted scaffolds shown in the examples are certainly qualitatively very similar, I did notice differences in details such as the orders of transitions (e.g., see the Monkey/RNN comparisons in Figure 3). Are there any situations where this ordering might be important?

LOOPER splits trajectories when they are unlikely to transition to each other in the diffusion map. The exact timing of this split is of course difficult to define, but it is likely to be accurate up to a few time points. However, in the biological data sets presented here we made use of delay embedding to uncover the dynamics of partially observed systems. Delay embedding invariably warps the timings. This, unfortunately, means that small timing differences are difficult to resolve. Direct testing would be required to judge whether the differences we see in the RNN vs monkey data are due to warping of time in the method.

In the fMRI dataset we explored the effect of model parameters on the timings of the trajectory splits (Figure 4C). This helps put statistical bounds on the time window during which the trajectories most likely split. Again, the precision of the timing depends heavily on the kinds of data that are used to build the model.

5) The references are incomplete. Some references are missing (e.g., the "?" in line 38), and the bibliography itself contains many references with incomplete citation information (e.g., refs 6, 12, 20, 43, 44, 50, 70, 72)

These have been fixed, thank you

Reviewer #2: The paper proposes an algorithm (LOOPER) for reduction of large-scale neuronal recordings to a finite, countable set of one-dimensional trajectories (the so-called "reduced model") that presumably encode stimuli and how they are internally processed by the brain. LOOPER is simulated and tested for encoding and reconstruction of several types of neuronal datasets: 1) noisy recurrent neural networks (RNNs) trained to perform a working memory task, 2) mean firing rates of single neurons in primates' prefrontal cortex during same working memory task, 3) calcium imaging data in C. elegans neurons, 4) visually evoked LFPs in mice, and 5) fMRI human data from a theory of mind task.

The algorithm seems very complicated, though thoroughly detailed in supplemental material. However, some of the algorithmic steps seemed to be added on the fly when the authors realized that they could not obtain the

expected results (e.g., adding an "external" node to ensure completeness of loops in certain neuronal recordings), while others seemed so complicated that their interpretation is hard to understand (e.g., subsequent construction of several matrices and distances). Numerous minimization steps were also included, and it is not clear to me how they guarantee a valid solution. Description of LOOPER in the main text should at least address these points and clarify them.

Thank you for raising this very important issue. Below we address your specific concerns.
Guarantees of valid solution:
LOOPER does not guarantee a valid solution. We now directly state this in the discussion section. Many of the admittedly regrettable complexities in the method are imposed by the noisy and incomplete observations of neuronal activity. Our goal here was to make a method that can be deployed on actual experimental observations. In comparatively simpler systems where a complete set of relevant variables can be measured in a large set of trials (e.g. noisy RNN) many of the processing steps are not required.

We have added a section to the main text (see below) pointing out that LOOPER does not guarantee a valid solution, and a section discussing the advancement of LOOPER over the *C. elegans* methodology. We hope that future work will simplify and optimize the methodology to extract stable trajectories from biological time series data. Nevertheless, we believe that the methodological and data processing complexities notwithstanding, we show that dynamics in many diverse biological and artificial systems can be well approximated by simple models that identify distinct trajectories and branching points that connect them. This is an important result and it is likely that specifics of the methodologies used to find such trajectories can be improved upon in the future.

Line 87
“LOOPER extends a similar methodology used in *C. elegans*. The *C. elegans* methodology used eigendecomposition of diffusion maps to find the primary cyclic mode in the data. However, we find that this technique fails in more complex systems. Eigendecomposition of the diffusion map only finds cyclic trajectories that are associated with a single phase velocity. While this strategy succeeded in *C. elegans,* in more complex systems this may not necessarily be the case. LOOPER addresses this concern by directly clustering the diffusion map to arrive at a model of one-dimensional trajectories. While these clustering and minimization steps inevitably add a level of complexity to the method and keep the method from guaranteeing any sort of optimality, we find that the method is effective at dealing with noisy experimental observations across a wide variety of model organisms and imaging types. Another important departure from the *C. elegans* data is that LOOPER is equipped to deal with trial data. Both LOOPER and the *C. elegans* method are naturally designed to work on continuous recordings, but with a simple modification, (Supplementary section **Extending LOOPER to work with trial data**) the method can also be applied to trial data where continuous recordings are not available. ”

Complexity of the method.
In *C. elegans* simple eigendecomposition of the diffusion map was sufficient to reconstruct the dynamics. However, when we attempted to apply similar methodologies to more complex systems such as those in the present paper, the method was not robust. This is why we added a number of processing steps.

The addition of the “terminal state” to the data is a simple way to make trial data continuous. It is not required by LOOPER, but is imposed onto us by the experimental design which did not record neuronal signals continuously. If data between trials was available to us, we would not need to introduce the terminal state or assume that all trials begin and end in the same state. Because we simply do not have access to the intertrial data we are forced to assume that all omitted data between trials belong to the same state. The only purpose of this terminal state is to facilitate the loop finding algorithm.

Line 1227
"Note that in reality all neuronal data is continuous, but experimental design may be such that parts of the recordings are left out. In order to maintain the assumption of continuity in the data we manually add a single hidden state to the LOOPER model. This hidden state represents all configurations of the system that are not observed in the recorded data, but none-the-less present in the actual neuronal activity."

It is not to say that LOOPER was not shown to successfully make some predictions. However, the results seem to apply only to bootstrapped and then trial-averaged data, and - apparently - not to the data themselves but rather to their projections on several principal components. A better description of the necessary steps on pre-processing of data used by LOOPER (including the justification) should be provided.

Standard machine learning techniques have great difficulty in classifying the fMRI data (even after bootstrapping and trial-averaging) (Figure 4D). LOOPER outperforms such techniques when applied to the same bootstrapped data, even if it is not on the single trial level. Similarly, LOOPER's reconstruction accuracy is comparable to HMM modeling on all the datasets presented (Figure 5). Taken together, this shows that even after simplifying the problem by averaging over bootstrapped trials, the problem remains challenging.

In *C. elegans* and RNNs we have (almost) complete direct observation of the neuronal activity of the system. In these cases bootstrapping or trial averaging was not necessary. We expect that as the observed experimental measure is increasingly abstracted away from the neuronal activity of the system the need for bootstrapping will increase. For the primate data, only a handful of neurons were recorded simultaneously on a given trial. We used the same bootstrapping over trials as in previous work on this dataset e.g. (Kobak 2016). Essentially, the bootstrapping involves concatenation of firing of different neurons that were recorded in similar trials (same stimulus conditions) but in different sessions.

Further, as shown in Figure 5 our model is predictive of not only PCs, but also individual neurons/ROIs. The PC projections are not an integral part of the method. Finally, as shown in Supplementary Figure S5, the model built on the trial-averaged monkey data is able to make predictions about neuronal activity even in single trials. Other methods (such as LFADs) are also able to make single trial predictions, but importantly these methods require almost 10 times as much data as was used in LOOPER. We expect that future improvements to the method will include machine learning approaches such as LFADs to extract a latent variable representation that will remove the need for trial averaging even on complex datasets.

Line 365
"Note that while preprocessing steps make use of PCA to reduce the spurious noise in the time series data, all predictions and reconstructions are on the level of individual channels (units in RNNs, neurons in \textit{C. elegans} and primates, electrode in mice and parcels in humans)."

Other questions/concerns are the following:

1) Figure 3 and related results: it seems that the projection of neural activity of the first three principal components already shows the 6 trajectories that LOOPER finds in the end. Does this bias the algorithm to find (again) those 6 trajectories? I mean, what is the advantage of running such a complex algorithm for this example in which the 6 different trajectories were rather obvious from a simple principal component analysis anyway?

The panels in Figure 3A and B are of the trial-mean subtracted data. The raw data projected onto the first PCs is shown in Supplementary Figure S9 and S10. In the raw data the separation of the trajectories is much less

clear. It is this raw data that was fed into LOOPER. Moreover (as you mention in your next comment), the real power of the LOOPER model is to find the points in the data in which trajectories split and merge, and to build quantitative models of the dynamics (Figure 5).

(Also, as a suggestion, it would be nice to keep same convention for solid lines versus dotted lines in panels A/C vs B/D)

Thank you. We have fixed the labels and ensured that all labeling conventions are consistent throughout the manuscript.

2) if I interpret the results correctly (say in fig 3 but also the other sections) it seems that the biggest advantage of obtaining the reduced model (one-dimensional trajectories) with LOOPER is to identify timing of changes in neural activity that predict changes in behavior. The "timing" of these events (when trajectories either split or merge) seem important and, most probably, could not be determined otherwise -- neither in the complicated phase space of original trajectories (neural activity) nor in the space of PCA projections. I think the authors should emphasize more this property of the algorithm. This may also explain why the correlation values for BOLD data in Fig 5 are much lower compared to the other neural data which have better temporal resolution.

Thank you. We absolutely agree that identifying the time when neuronal trajectories branch and merge is a major advantage of our methodology that we regret not emphasizing this appropriately in the original submission.
We have clarified the section that discusses this point in the paper.

Line 263
“One advantage of LOOPER over other heuristic techniques is that the computational scaffold extracted by LOOPER is quantitatively accurate. This means that the timings of algorithmic events (splits and merges of the trajectories) are able to be approximated. The exact timings of these events would still require direct experimentation to confirm, as the LOOPER model assumes a level of stochasticity and makes use of state space reconstruction that both lead to the warping of temporal information.”

3) line 286 (and other places): "We then projected experimental observations from a non-overlapping validation dataset into the model space […]"
How exactly is this done?
Also, it is not clear to me how the reconstruction of data from the simplified model was done.

Projection into the model state is done by finding the state in the model that is most similar to the position in state space of the validation data point. The details on this projection are given in the Supplementary section “Validation of computational scaffold”. We have added a call out to the relevant section in the main text and moved this section to the Methods.

4) for some of the results, training and testing data depend on each other (e.g., Fig 4) raising questions about the strength of the reported results

The LOOPER model is not trained on the PCA of both the training and validation data, but only the PCA of the training data. The joint PCA modification was only applied to the SVM model. We have added a sentence clarifying this point.

Line 318
“Thus, to reduce the degrees of freedom we projected the preprocessed fMRI data onto principal components analysis (PCA) space before averaging (20 components, 71\% variance). Note that LOOPER did not require this preprocessing step. Preprocessing the data with PCA did not improve classification performance significantly (Figure~\ref{Fig:4}D, orange). However, when the data from the validation dataset was added to the calculation of PCA, the classification performance of the SVM model improved dramatically (Figure~\ref{Fig:4}D yellow, p-value $<$0.001). Again, LOOPER was trained on just the raw parcel data.”

5) a panel in Figure 5 is missing (Fig 5K)

The *C. elegans* data has no associated task, and so we cannot decode the task condition. We have modified the figure to make this more clear.

Figure 5 legend
“Note that the mouse data has no associated task and so is left blank.”

6) there are several typos in the paper, including in the text of the figures themselves (e.g., Fig 4 "separation"; caption of Fig4, - p value; Fig 5 "initial trace"; etc). Some references are missing (line 38).

We have fixed these, thank you.
